# Involvement of the habenula in the pathophysiology of autism spectrum disorder

**DOI:** 10.1038/s41598-021-00603-0

**Published:** 2021-10-27

**Authors:** Jürgen Germann, Flavia Venetucci Gouveia, Helena Brentani, Saashi A. Bedford, Stephanie Tullo, M. Mallar Chakravarty, Gabriel A. Devenyi

**Affiliations:** 1 University Health Network, 399 Bathurst Street, Toronto, ON Canada; 2 Cerebral Imaging Centre, Douglas Mental Health University Institute, McGill University, Montreal, QC Canada; 3 Neuroscience and Mental Health, Hospital for Sick Children Research Institute, Toronto, ON Canada; 4 Department of Psychiatry, University of Sao Paulo, Medical School, São Paulo, São Paulo Brazil; 5 National Institute of Developmental Psychiatry for Children and Adolescents, CNPq, São Paulo, São Paulo Brazil; 6 Autism Research Centre, Department of Psychiatry, University of Cambridge, Cambridge, United Kingdom; 7 Integrated Program in Neuroscience, McGill University, Montreal, QC Canada; 8 Department of Biomedical Engineering, McGill University, Montreal, QC Canada; 9 Department of Psychiatry, McGill University, Montreal, QC Canada

**Keywords:** Autism spectrum disorders, Brain, Social behaviour

## Abstract

The habenula is a small epithalamic structure with widespread connections to multiple cortical, subcortical and brainstem regions. It has been identified as the central structure modulating the reward value of social interactions, behavioral adaptation, sensory integration and circadian rhythm. Autism spectrum disorder (ASD) is characterized by social communication deficits, restricted interests, repetitive behaviors, and is frequently associated with altered sensory perception and mood and sleep disorders. The habenula is implicated in all these behaviors and results of preclinical studies suggest a possible involvement of the habenula in the pathophysiology of this disorder. Using anatomical magnetic resonance imaging and automated segmentation we show that the habenula is significantly enlarged in ASD subjects compared to controls across the entire age range studied (6–30 years). No differences were observed between sexes. Furthermore, support-vector machine modeling classified ASD with 85% accuracy (model using habenula volume, age and sex) and 64% accuracy in cross validation. The Social Responsiveness Scale (SRS) significantly differed between groups, however, it was not related to individual habenula volume. The present study is the first to provide evidence in human subjects of an involvement of the habenula in the pathophysiology of ASD.

## Introduction

**Figure 1:**
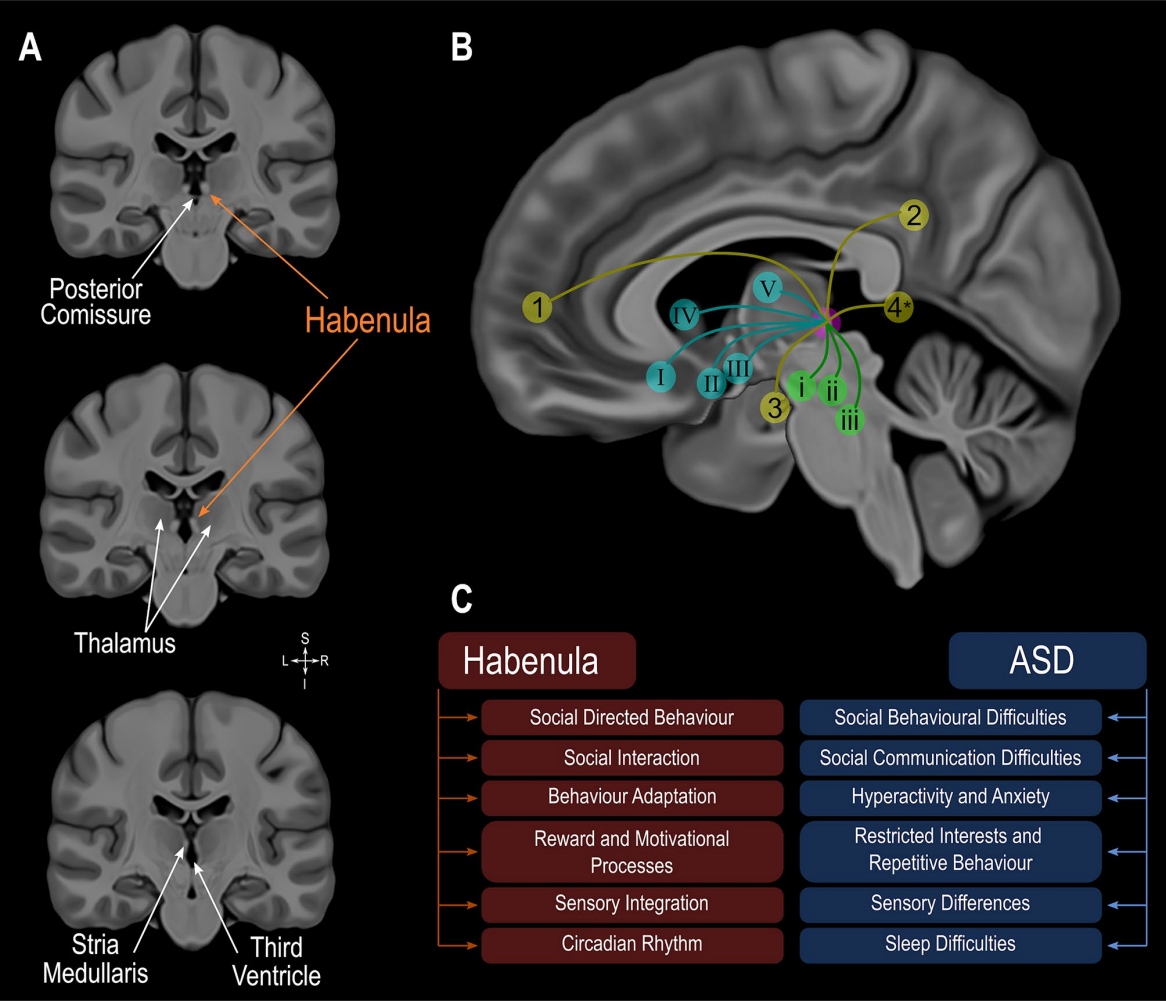
Habenula anatomy, boundaries and connections displayed using a high-resolution, high contrast template by Neudorfer and colleagues^50^. (**A**) Coronal slices illustrating the location of the Habenula, a structure appearing bright (hyperintense) on T1 weighted magnetic resonance images, surrounding structures and its boundaries. (**B**) Diagram illustrating the connectivity of the habenula. Cortical regions in yellow: (1) medial prefrontal cortex; (2) cingulate gyrus; (3) hippocampus and parahippocampal gyrus; (4) posterior insula (*estimated location). Subcortical regions in blue: (I) basal forebrain; (II) hypothalamus; (III) nucleus basalis of Meynert; IV, basal ganglia; V, thalamus. Brainstem regions in green: (i) ventral tegmental area; (ii) substantia nigra; (iii) periaqueductal grey—raphe nuclei. (**C**) Functions that the Habenula is critically involved in and differences found in autism spectrum disorder. ASD: autism spectrum disorder.

The habenula is a small phylogenetically preserved epithalamic structure that plays a key role in the control of the monoaminergic system^1,2^. It is divided into lateral and medial parts based on characteristic cytoarchitectonic and connectivity patterns. Through its rich widespread connections, especially to the hypothalamus, limbic areas and brainstem nuclei (illustrated in Fig. 1) the habenula is implicated, among others, in social interaction, reward processing, behavioral adaptation, sensory integration and circadian rhythm (Fig. 1)^1–8^.

Core Autism Spectrum Disorder (ASD) symptoms are social communication difficulties, restricted interests, repetitive behaviors. Furthermore, altered sensory perception as well as mood and sleep disorders are frequently identified in ASD subjects (Fig. 1)^9,10^. ASD is a neurodevelopmental disorder that is 3 to 4 times more frequently diagnosed in males than females^11,12^. Affected individuals usually show symptoms of the disorder as early as 12 to 18 months of age^9,13–15^ and multiple differences in functional and morphological brain phenotype have been reported in ASD^15–23^. In particular studies demonstrate that brain development is altered in individuals with ASD as exemplified by deviations from normal developmental trajectories in regional cortical thickness, surface area and structure volume^16–18,23–25^. Some of these brain changes are related to individual characteristics such as symptom severity and sex. Specific differences associated with ASD have been found in key areas of the brain that underlie processing of social cues, areas critically involved in understanding others such as the posterior superior temporal sulcus area, fusiform gyrus and amygdala^26–30^.

The habenula is prominently involved in the processing of social information and the regulation of social behavior^5,8^. It is, furthermore, a key region for reward processing and critically involved in a number of depressive behaviours^1,6,31,32^. In addition, studies have implicated the habenula in bipolar disorder (BD)^33–35^, obsessive–compulsive disorder (OCD)^36,37^ as well as schizophrenia^38,39^ and altered habenula volume has been reported in subjects diagnosed with BD and schizophrenia^40,41^. Previous studies have reported altered amygdala volume in subjects with ASD compared to age matched controls^25,42^. While subsequent research showed these volume differences might only be apparent at certain ages, these point to an altered developmental trajectory of this important social brain area in ASD^21,23,25,42,43^. So while neuroimaging and brain stimulation techniques have provided some insight into the role of the habenula in psychiatric disorders further research is warranted to obtain a deeper understanding of the neurocircuitry of social behaviour and relate it to the differences found in human disorders^6,40,41,44–49^. Similar to the amygdala findings, altered habenula volume across development might be found in subjects with ASD compared to age matched controls like it has been described in BD^41^.

Several preclinical studies have investigated the relationship between habenula and autism phenotypes by exploring behaviour, genetics, electrophysiology and functional neuroanatomy of wild-type and transgenic animals. A transcriptomic-anatomic analysis of the rodent habenula revealed a large collection of enriched genes associated with autism-related transcripts^51^. Electrophysiological recordings of habenular neurons detected transient and high frequency T-type Ca^2+^ channel-mediated firing, a channel implicated in ASD^52^, altered ion channel function, abnormal firing pattern and hypo-excitability^53^. The inhibition of the lateral habenula in juvenile and adult rats by microinjection of GABA-A and GABA-B receptor agonists markedly reduces social play behaviour (juvenile rats) and behavioural flexibility (adult rats), suggesting a critical role of the habenula in processing social information and selecting behavioural actions under challenging cognitive or emotional situations, differences also seen in ASD^8,54^. Interestingly, reduction in oxytocin innervation in the lateral habenula, a neuropeptide closely involved in social bonding, is thought to be the underlying mechanism of social impairment in the Mecp2-null mouse model of Rett syndrome^55^.

In line with these findings, a study using excitatory designer receptor exclusively activated by designer drugs (DREADD) showed that frontal cortex activation suppressed social behaviour via activation of lateral habenula neurons; inhibition of these neurons prevented the social behavioural deficits observed after frontal cortex activation^5^. Integrity of the fiber tracts connecting the habenula to the midbrain tegmentum were also described as critical for social behaviour as observed in double Tg mice designed to express alterations in neural crest-derived cells^56^. Furthermore, altered temporal patterns in the mesolimbic/habenular reward circuit have been described in the fmr1 knockout rat model of Fragile X syndrome and associated with the abnormal behavioural response in odor-investigation paradigms^57^.

Thus, an involvement of the habenula in the neurobiology of ASD is plausible but has yet to be demonstrated in humans. Here, we investigated the hypothesis that the habenula plays a role in ASD by analyzing morphometric habenula characteristics in a large cohort of ASD subjects (220 subjects; 184 males) and age matched typically developing controls (TDC; 303 subjects; 213 males) from the Autism Brain Imaging Data Exchange (ABIDE) repository^58,59^, with an age range spanning from early childhood to adulthood (6 to 30 years; Table 1). A complete Social Responsiveness Scale (SRS)^60^ score was available for all subjects, ASD and TDC. The SRS is a rating scale filled out by a next of kin or caregiver to quantitatively measure autistic traits and is a widely used reliable as a screening tool for children, adolescents and adults^61–63^. Possible effects of sex and individual SRS score on habenula volume were investigated.

**Table 1:** Demographics.

Site	n	ASD	TDC	Average total SRS	Average full IQ	Average total brain volume (mm^3^)	Average bilateral habenula volume (mm^3^)
		n (M/F)	n (M/F)				
		Age mean ± SD (range)	Age mean ± SD (range)				
University of Leuven	52	23 (21/2)	29 (25/4)	ASD: 82.522 ± 30.133	ASD: 114.095 ± 12.429	ASD: 3,035,573.715 ± 294,170.657	ASD: 26.245 ± 4.944
		17.79 y ± 4.34 y (12–29 y)	18.32 y ± 5.11 y (12–29 y)	TDC: 30.345 ± 22.946	TDC: 112.957 ± 12.323	TDC: 3,051,012.325 ± 283,960.252	TDC: 26.227 ± 4.834
New York University	133	65 (59/6)	68 (55/13)	ASD: 91.831 ± 27.939	ASD: 106.769 ± 18.068	ASD: 3,000,324.962 ± 390,755.495	ASD: 27.930 ± 5.901
		14.13 y ± 6.32 y (6–30 y)	11.37 y ± 3.04 y (6–18 y)	TDC: 22.696 ± 13.036	TDC: 113.652 ± 15.394	TDC: 2,955,228.120 ± 261,652.335	TDC: 26.619 ± 5.759
University of Utah School of Medicine	52	27 (27/0)	25 (25/0)	ASD: 84.148 ± 32.884	ASD: 100.852 ± 14.114	ASD: 3,060,374.104 ± 285,066.478	ASD: 27.922 ± 5.279
		20.48 y ± 3.98 y (14–29 y)	20.75 y ± 5.25 y (10–30 y)	TDC: 16.160 y ± 12.733	TDC: 114.280 ± 14.513	TDC: 3,172,042.086 y ± 344,710.406	TDC: 25.596 y ± 6.764
Yale Child Study Center	24	12 (8/4)	12 (7/5)	ASD: 95.500 ± 29.998	ASD: 94.750 ± 19.377	ASD: 2,902,912.449 ± 310,118.915	ASD: 27.083 ± 4.144
		13.12 y ± 3.28 y (7–18 y)	14.75 y ± 1.91 y (11–18 y)	TDC: 27.833 ± 24.439	TDC: 98.667 ± 13.780	TDC: 2,912,640.938 ± 298,063.917	TDC: 26.333 ± 4.355
George Town University	21	7 (6/1)	14 (9/5)	ASD: 85.571 ± 42.308	ASD: 114.857 ± 9.737	ASD: 2,925,413.917 ± 161,283.395	ASD: 29.857 ± 5.113
		11.55 y ± 0.74 y (10–13 y)	10.95 y ± 1.83 y (8–14 y)	TDC: 19.571 ± 19.575	TDC: 117.214 ± 13.227	TDC: 2,904,958.613 ± 336,317.471	TDC: 21.071 ± 3.772
Indiana University	16	10 (9/1)	6 (4/2)	ASD: 88.700 ± 38.006	ASD: 114.000 ± 16.083	ASD: 3,217,222.049 ± 279,441.641	ASD: 27.817 ± 5.060
		21.80 y ± 4.02 y (17–28 y)	22.17 y ± 2.32 y (20–25 y)	TDC: 48.500 ± 12.373	TDC: 114.500 ± 5.541	TDC: 3,106,244.547 ± 253,565.846	TDC: 23.896 ± 2.144
Kennedy Krieger Institute	107	18 (9/9)	89 (52/37)	ASD: 95.222 ± 25.915	ASD: 108.722 ± 13.702	ASD: 2,896,036.193 ± 293,025.042	ASD: 25.782 ± 4.218
		10.75 y ± 1.83 y (8–13 y)	10.53 y ± 1.31 y (9–13 y)	TDC: 16.531 ± 10.583	TDC: 119.313 ± 10.322	TDC: 2,823,588.809 ± 266,549.718	TDC: 25.806 ± 3.906
Oregon Health and Science University	65	27 (22/5)	38 (18/20)	ASD: 91.778 ± 26.536	ASD: 106.111 ± 17.190	ASD: 3,101,614.595 ± 389,037.345	ASD: 25.652 ± 5.700
		12.07 y ± 2.02 y (8–15 y)	10.34 y ± 1.65 y (8–14 y)	TDC: 24.474 ± 17.573	TDC: 116.816 ± 11.613	TDC: 2,980,955.747 ± 300,032.667	TDC: 26.087 ± 3.959
San Diego State University	37	21 (15/6)	16 (14/2)	ASD: 100.571 ± 23.756	ASD: 97.429 ± 14.678	ASD: 3,000,965.011 ± 291,295.616	ASD: 27.143 ± 6.142
		13.71 y ± 3.06 y (8–18 y)	14.15 y ± 2.79 y (10–18 y)	TDC: 17.938 ± 10.951	TDC: 103.313 ± 9.286	TDC: 3,046,140.478 ± 270,841.463	TDC: 25.250 ± 4.553
University of California Davis	16	10 (8/2)	6 (4/2)	ASD: 75.800 ± 34.428	ASD: 104.800 ± 11.243	ASD: 3,308,879.166 ± 373,647.297	ASD: 28.600 ± 6.569
		15.23 y ± 2.05 y (12–18 y)	15.88 y ± 1.14 y (14–17 y)	TDC: 10.667 ± 6.593	TDC: 114.167 ± 12.416	TDC: 114.167 ± 12.416	TDC: 28.500 ± 6.058
Total	523	220 (184/36)	303 (213/90)	ASD: 90.150 ± 29.551	ASD: 105.316 ± 16.464	ASD: 3,031,526.311 ± 344,653.350	ASD: 27.263 ± 5.490
		14.99 y ± 5.34 y (6–30 y)	12.94 y ± 4.64 y (6–30 y)	TDC: 21.868 ± 16.026	TDC: 113.652 ± 12.977	TDC: 2,976,081.507 ± 302,749.991	TDC: 25.657 ± 4.849

*ASD* autism spectrum disorder, *TDC* typically developing controls, *IQ* intelligence quotient, *SRS* social responsiveness scale.

This investigation was performed using the fully automated segmentation of the habenula tool in MAGeTbrain^41^. This tool has been shown to be reliable to evaluate habenula volume in large datasets that includes subjects with a wide age range spanning childhood to late adulthood and using distinct MRI acquisition parameters^41^. MAGeTbrain is a well established methodology that produces a segmentation for each subject using a multi-atlas voting procedure via image registration. Segmentations from each atlas are propagated to create a large number of candidate segmentations that are fused using majority vote, a process that reduces bias and averages registration errors while allowing for the neuroanatomical variability of the subjects to refine each individual subject’s final segmentation^64–66^.

## Results

**Figure 2:**
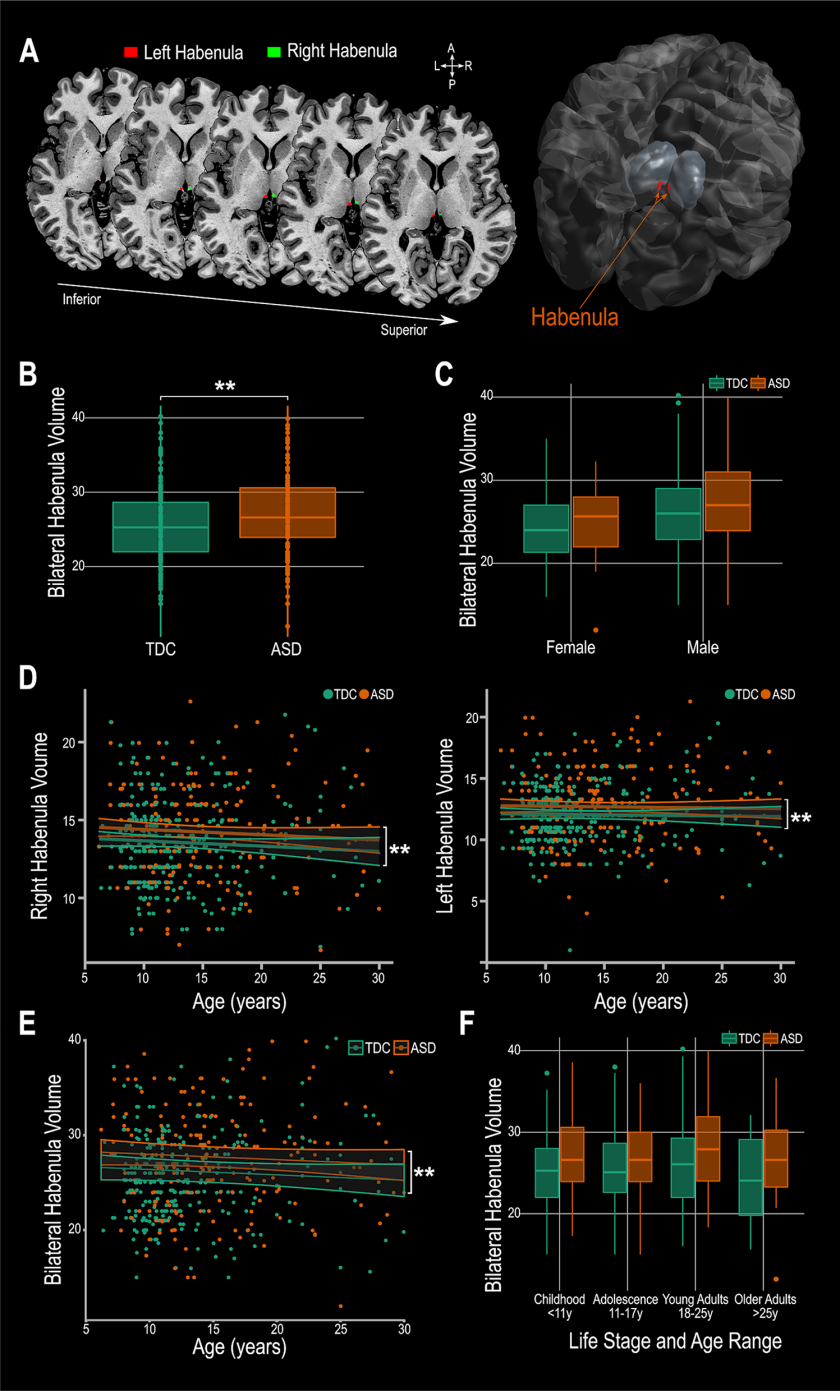
Habenula volume differences found in ASD. (**A**) Example MAGeTBrain habenula segmentation on a T1 weighted magnetic resonance image illustrated on the axial plane and 3D reconstruction of the habenula. (**B**) Bilateral habenula volume is greater in ASD compared to TDC. (**C**) The group effect (bilateral habenula larger in ASD vs TDC) is independent of sex. (**D**) There is no effect or laterality; the habenula is larger in ASD compared to TDC in the right and in the left hemisphere. This effect is apparent across the entire age range tested (**E**) within all age groups (**F**). ** indicates p ≤ 0.01. ASD: autism spectrum disorder subjects; TDC: typically developing controls.

The morphometry (volume) of the left and right habenula of each subject was obtained through automatic segmentation (Fig. 2A)^41,64^. To ensure that the automated segmentation process can be reliably applied in autistic subjects, in particular focusing on the pediatric sample, the left and right habenula of 24 randomly selected brains (8 from the childhood sample (< 11 years); 8 from the adolescence sample (12–17 years); 8 from the young adults sample (18–25 years); 48 habenula altogether) were manually segmented. The Dice similarity coefficients (DSC) are: childhood sample: DSC left habenula 0.82, DSC right habenula 0.82 (range 0.73–0.88); adolescence sample: DSC left habenula 0.82, DSC right habenula 0.83 (range 0.70–0.91); young adult sample: DSC left habenula 0.81, DSC right habenula 0.80 (range 0.72–0.89) overall: DSC left habenula 0.82, DSC right habenula 0.82 (range 0.70–0.91). These results are similar to those reported in the previous validation^41^ and confirm that the habenula can be reliably segmented in this patient population using this method.

Comparing total bilateral habenula volume (co-varied with age, sex and total brain volume with the study site used as a random intercept effect) we found that ASD subjects have significantly larger bilateral habenula volumes compared to TDC (ASD subjects: 27.1 mm^3^ ± 5.3; TDC: 25.5 mm^3^ ± 4.5; t = 3.28, p = 0.001; Fig. 2B). Habenula volume did not differ between males and females (Fig. 2C). This significant volume difference is apparent in both the right (t = 2.89, p = 0.004) and left (t = 2.76, p = 0.006) habenula (Fig. 2D) and across the entire age range studied (6 to 30 years; Fig. 2E,F).

**Figure 3:**
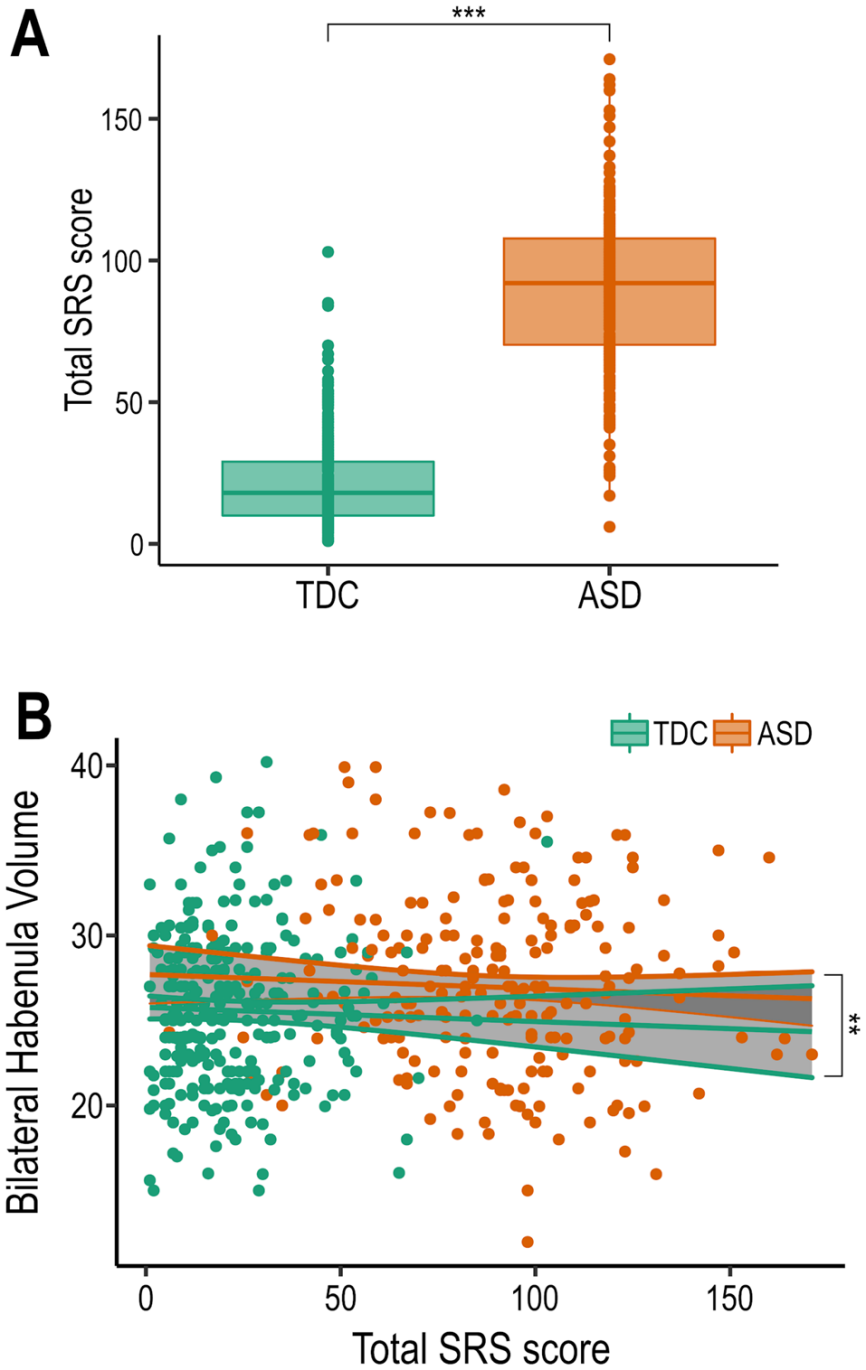
(**A**) Main effect of diagnosis on SRS score. (**B**) No significant interaction of Habenula volume and SRS score in either group. ** indicates p ≤ 0.01; *** indicates p ≤ 0.001. SD: autism spectrum disorder subjects; TDC: Typically developing controls.

The SRS scores are significantly different between groups (t = 32.23, p < 2e−16) (Fig. 3A), as expected as it is used as a screening tool, individual SRS scores, however, are not related to individual habenula volumes beyond diagnosis (larger in ASD) (Fig. 3B). Thus individual autistic traits as quantified by the SRS were not found to be related to individual bilateral habenula volume within either group (ASD or TDC).

Support-vector machine modelling demonstrates that habenula volume differentiates between groups (model using age, sex and bilateral habenula volume) and can claissfy ASD as compared to TDC with 85% accuracy. The accuracy dropped to 64% in the cross validation (Table 2).

**Table 2:** SVM classifier and cross validation.

Support vector machine (SVM) classifier			
	ASD	Control	Total
Predicted ASD	256	43	299
Predicted control	47	260	307
Total	303	303	606
Accuracy: 85%; Sensitivity: 85%; Specificity: 86%			

Four fold cross validation of SVM classifier			
	ASD	Control	Total
Predicted ASD	191	108	299
Predicted control	112	195	307
Total	303	303	606
Accuracy: 64%; Sensitivity: 63%; Specificity:64%			

A support vector machine classifier using individual age, sex and bilateral habenula volume as input is able to distinguish between ASD and TDC control subjects with 85% accuracy using a balanced dataset created by adding ASD subjects randomly picked from the original 220. The accuracy drops to 64% in a fourfold cross validation where for every quarter of the original dataset, the SVM is trained on the remaining three quarters and applied to the unseen data.

ASD, autism spectrum disorder; TDC, typically developing controls; SVM support vector machine.

## Discussion

Investigating habenula volume in a large cohort of ASD and TDC subjects spanning in age from childhood to young adulthood we found that habenula volume is larger in subjects diagnosed with ASD across all ages. While studies using preclinical models have provided evidence of an involvement of the habenula in autism phenotypes^8,51–57^ these findings in human subjects provide further evidence of the involvement of the habenula in the pathophysiology of ASD. The habenula volume difference did not show any evidence of being the product of an altered developmental trajectory. Similarly, there was no evidence of an effect of sex or symptom severity as measured by the individual SRS score. These findings are distinctly different compared to other morphological differences associated with ASD and in particular are unlike the abnormal developmental time course, sex differences and effects of symptom severity described in alterations of amygdala volume associated with ASD^20,23,42,43^. Likewise, a previous study investigating habenula volume differences associated with schizophrenia and BD found that habenula volume differences were only apparent in certain age ranges^41^.

While T1w contrast does not allow one to discern the underlying cause of these volume differences (e.g. increased dendrites, microglia, angiogenesis, neuroglia) previous work in animals showed that social deficits are associated with increased habenula activity and that experimentally inhibiting habenula activity improves social behaviour^5^. The habenula has been identified as the central structure mediating the reward value of social interactions and shown to be a key region for adapting behavioural strategies, integrating both internal (previous reward experience) and external (sensory input) information to initiate necessary behavioural adjustments^2,4^. Furthermore, an optimal habenula function is necessary for flexible behavioural adjustments^2,4^, sensory processing^2,6^, motivational processes^1,2^ and regulation of circadian rhythms^3^ all domains where differences are found in ASD^9,10^ (e.g. social communication difficulties, restricted interests and repetitive behaviors, altered sensory perception and mood and sleep disorders; Fig. 1).

This study has a number of limitations. While the rigorous quality control of the individual MRI scans is necessary to ensure that the findings are reliable, subjects excluded due to quality control issues were more likely to be from the ASD group, significantly younger, had lower full IQ and higher symptom severity scores and were more likely to be male (Bedford et al. Supplementary Table S5^16^). Future tools might allow correction of motion artefacts and will eliminate potential biases in the data exclusion. The study covered a large age range, but did not include subjects from early childhood. ASD is typically diagnosed in that period and further studies are needed to investigate the early trajectory of habenula volume and a possible relationship of habenula volume with autistic symptoms and diagnosis. Furthermore, while the study included female ASD subjects, the group was relatively small.

Despite these limitations, the robust finding of increased habenula volume in ASD compared to TDC subjects provides the first evidence in human subjects of an involvement of the habenula in some aspect of the pathophysiology of ASD. The fact that there is a strong effect of diagnosis indepedent of age, sex or symptom severity as assessed by the SRS score might point to the habenula being implied in a broader range of behavioral symptoms, beyond the classic deficits of social behaviour and social interaction. Further studies investigating neurotransmitters, metabolites, connectivity patterns and neurochemical binding are necessary to unravel the neurobiological mechanisms underlying the involvement of the habenula in ASD.

## Materials and methods

### Subjects

Habenula volumes were estimated using T1-weighted magnetic resonance images (MRI) scans of subjects from the Autism Brain Imaging Data Exchange (ABIDE) repository^58,59^. ABIDE is a large-scale, publicly available multi-site database providing MRI scans as well as behavioral data from individuals with autism spectrum disorder and typically developing controls. For detailed demographics, imaging acquisition parameters, local institutional review boards and written informed consent, please visit (https://fcon_1000.projects.nitrc.org/indi/abide/). Inclusion criteria were motion and scan quality as previously described^16^, complete Social Responsiveness Scale scores^60^, sufficient data to model the developmental trajectories in ASD subjects and TDC. The SRS is a rating scale filled out by a next of kin or caregiver to quantitatively measure autistic traits and is a widely used reliable screening tool for children, adolescents and adults^61–63^. This resulted in a final dataset of 220 ASD subjects (184 males) and 303 TDC (213 males) ages 6 to 30 years (Table 1).

### Image processing, MAGeTbrain segmentation and validation

The fully automated segmentation of the Habenula has been previously validated and shown to be a reliable toll for the assessment of habenula volume in large datasets, across a wide age range spanning from childhood to late adulthood, and using distinct MRI acquisition parameters^41^. The dataset used to establish validity and reliability included 103 scans (out of a total 356) of children and adolescents aged 4 to 17 years of age. All automatic segmentations were rigorously visually inspected to ensure successful segmentation^41^. The validation included comparing the automatic segmentations to manually segmented habenulas (n = 30 (2 × 15 subjects)). These were randomly sampled and thus included approximately 10 manually segmented habenulas (~ 1/3 of scans) of subjects younger than 18 years. Reliable segmentation (as compared to manual segmentation for two raters) was demonstrated across individual habenulas ranging from in volume from 7 to over 20 mm^3^ and ages from 4 to 50 years demonstrating that reliable automatic segmentation is independent of habenula size and subject age. Image processing and quality control of the images was performed similar to previously described^16^. The images were pre-processed using iterative non-uniformity correction and skull-stripped (https://github.com/CoBrALab/minc-bpipe-library). In-scanner subject motion may bias neuroanatomical measures derived from the anatomical images^67–69^, and might lead to erroneous morphological group differences being found. All images were therefore rigorously quality controlled by two independent raters (SAB, and either ST or MMC). The detailed QC method and examples can be found in the main text and supplement of Bedford and colleagues^16^. Only scans of high quality were included in the study (see exclusion criteria above) to ensure the reliability of the morphological measurements. The Multiple Automatically Generated Templates (MAGeT) brain segmentation algorithm was used to segment the habenula (https://github.com/CoBrALab/MAGeTbrain)^41,64^. MAGeTbrain was designed, from the onset, to improve the segmentation accuracy and robustness of atlas-based segmentation techniques. It has been shown to provide reliable and accurate segmentations of subcortical structures as well as hippocampal subfields and cerebellar lobules using only T1w image^65,70–72^. MAGeTbrain employs label propagation to produce individual segmentations using five segmented high-resolution atlases. It employs image registration and habenula segmentation is aided by the high local contrast provided by the third ventricle and the thalamus (Fig. 1)^41^. These atlases are then, again using image registration, propagated to 21 template images selected from the input dataset. In doing so a large number (5 × 21 = 105) of candidate segmentations is created, which are fused using majority vote to derive a final segmentation for each subject. Using this template library has two advantages: it helps reduce atlas bias and it reduces registration errors by averaging^65^. To ensure accurate segmentation, the resulting individual habenula segmentation of each subject was visually inspected independently by two raters (JG and FVG) in 3D overlaid onto the individual T1-weighted MRI image using DISPLAY (https://www.mcgill.ca/bic/software/minc/minctoolkit). Segmentation of the habenula was inspected in continuous slices in all three planes and rated as successful (correct location and extend in all dimensions) or failed segmentations (over- or under segmentation). Both raters agreed on the quality of segmentation in all cases.

Furthermore, to confirm that the habenula can be reliably segmented using the automated method in this autistic patients population the left and right habenula of 24 randomly selected brains were manually segmented. The 24 brains were taken from the childhood sample (< 11 years), the adolescence sample (12–17 years) and the young adults sample (18–25 years) (8 each, 48 habenula altogether). The Dice similarity coefficients (DSC) were calculated and used for validation. Previous work demonstrated the reliability with average DSC scores between 0.78 and 0.84^41^.

### Statistical analysis

The lme4 (version 1.1-21), e1071 (version 1.7-3) and lmerTest (version 3.1.1) packages in R (version 3.6.1; https://www.r-project.org) were used to perform the statistical analysis. Bilateral habenula volumes (left habenula + right habenula) were calculated and used for subsequent analysis. A linear mixed effect model corrected for age, sex and total brain volume with the site used as a random intercept effect was used to test for a possible effect of diagnosis on individual habenula volume: linear mixed effect model = “bilateral habenula volume” ~ “age” + “sex” + “total brain volume” + “group (ASD or TDC)” + (1|”study site”).

Individual total brain volume was derived from the brain mask created during preprocessing and describes the volume of the cerebrum, cerebellum, brainstem and ventricles. As similar linear mixed effect model corrected for age, sex and total brain volume with the site used as a random intercept effect was used to test for a possible effect of individual SRS score on individual habenula volume within each group (ASD or TDC) separately.

Support-vector machine (SVM) learning was used to interrogate the value of habenula volume in predicting diagnosis. To this end age, sex and bilateral habenula volume were used as predictors using a balanced dataset of 303 observations for both AD and TDC; additional observations for the ASD group were created by random sampling with replacement. A fourfold cross validation was used to validate the model.

## Data Availability

The dataset analysed during the current study (Autism Brain Imaging Data Exchange, ABIDE I & ABIDE II) is publicly available at https://fcon_1000.projects.nitrc.org/indi/abide/. The tool used in this study for automatic segmentation of the habenula is freely available at https://github.com/CoBrALab/MAGeTbrain. The processed data are available from the corresponding author upon reasonable request.
